# Canine neuroanatomy: Development of a 3D reconstruction and interactive application for undergraduate veterinary education

**DOI:** 10.1371/journal.pone.0168911

**Published:** 2017-02-13

**Authors:** Hazel Raffan, Julien Guevar, Matthieu Poyade, Paul M. Rea

**Affiliations:** 1 Laboratory of Human Anatomy, Thomson Building, School of Life Sciences, College of Medical, Veterinary and Life Sciences, University of Glasgow, United Kingdom; 2 Digital Design Studio, Glasgow School of Art, Glasgow, United Kingdom; 3 School of Veterinary Medicine, College of Medical, Veterinary and Life Sciences, University of Glasgow, Glasgow, United Kingdom; University of Bari, ITALY

## Abstract

Current methods used to communicate and present the complex arrangement of vasculature related to the brain and spinal cord is limited in undergraduate veterinary neuroanatomy training. Traditionally it is taught with 2-dimensional (2D) diagrams, photographs and medical imaging scans which show a fixed viewpoint. 2D representations of 3-dimensional (3D) objects however lead to loss of spatial information, which can present problems when translating this to the patient. Computer-assisted learning packages with interactive 3D anatomical models have become established in medical training, yet equivalent resources are scarce in veterinary education. For this reason, we set out to develop a workflow methodology creating an interactive model depicting the vasculature of the canine brain that could be used in undergraduate education. Using MR images of a dog and several commonly available software programs, we set out to show how combining image editing, segmentation and surface generation, 3D modeling and texturing can result in the creation of a fully interactive application for veterinary training. In addition to clearly identifying a workflow methodology for the creation of this dataset, we have also demonstrated how an interactive tutorial and self-assessment tool can be incorporated into this. In conclusion, we present a workflow which has been successful in developing a 3D reconstruction of the canine brain and associated vasculature through segmentation, surface generation and post-processing of readily available medical imaging data. The reconstructed model was implemented into an interactive application for veterinary education that has been designed to target the problems associated with learning neuroanatomy, primarily the inability to visualise complex spatial arrangements from 2D resources. The lack of similar resources in this field suggests this workflow is original within a veterinary context. There is great potential to explore this method, and introduce a new dimension into veterinary education and training.

## Introduction

The use of modern technology and virtual reconstructions has been commonly used in anatomical education within the medical curriculum. Indeed, since the development of the creation of the first digital anatomical teaching materials like the Visible Human Project (VHP) [1], there has been an explosion onto the market of products and tools for educational use [2–13]. Indeed, a wide variety of imaging methodologies and presentation of interactive material has been developed to aid the educator and the student alike over recent years [5, 6, 14, 15].

However, in the field of veterinary medicine and surgery, there has been a serious lack of detailed anatomical models similar to that of the VHP. Whole and partial datasets certainly have been developed using similar techniques to the VHP like a frog [16], a rat brain [17], and an equine metacarpophalangeal joint [18]. Yet, despite these attempts, few have been fully exploited for educational purposes and were mostly lacking the level of detail required by veterinary students. The most notable endeavour was that of the Visible Animal Project (VAP) [19], where a high-resolution 3D database of the dog trunk was created with the intention of being used as the basis of further interactive reconstructions. While the quality of this model certainly exceeded those that had gone before it, it lacked detail of the cranial anatomy, in particular the vasculature, a region that is notoriously difficult for students to understand.

More recently, non-invasive methods of visualising cranial anatomy have been described, based on using image segmentation of Magnetic Resonance Imaging (MRI) and Computed Tomography (CT) data alone [20,21]. While it was not the intention of these studies to create interactive learning environments for veterinary students, it is clear that the methods described could be harnessed for such a task. More recently Manson et al. [4] had devised a workflow methodology for the creation of an interactive training and educational anatomy application of the cerebral ventricles and flow of cerebrospinal fluid in humans. This shows the ease at which these packages could be created for a variety of different specialties and professionals.

With such a lack of digital resources in veterinary anatomy for education and training [22–27], and the relative difficulty that cranial neurovasculature poses to the veterinary student, there is an obvious need to translate this into the undergraduate veterinary curriculum. Given the existing shortages, there is great potential to create and build an interactive, anatomically correct 3D model of the vasculature of the canine brain, a resource that would undoubtedly be a valuable tool in veterinary education.

As such, we set out to use MRI and CT scanning, and used the images with widely available software packages to create a fully interactive educational and training package of the vasculature of the canine species. This is hoped to aid the understanding of this complex area of anatomy for students of veterinary medicine and surgery. As such, this workflow methodology created here can be the basis for further development in this area of research to aid undergraduate (and postgraduate) educators in developing validated digital training materials for their students.

## Materials and methods

**Fig 1:**
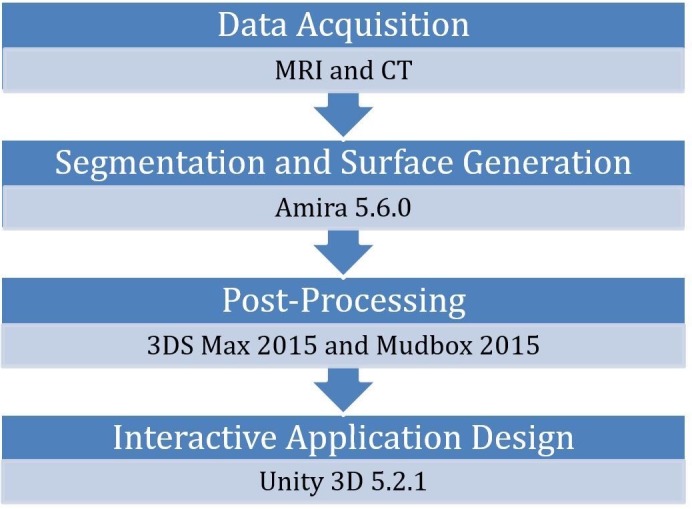
Visualisation pipeline used to create a 3D reconstruction and interactive application.

A total of 11 magnetic resonance images (MRI) were used from a 7-year-old Cavalier King Charles Spaniel in dorsal recumbent position, as part of advanced imaging diagnostic investigations at the University of Glasgow’s Small Animal Hospital, and were in the standard DICOM file format. This patient was clinically normal, and not formally investigated for Chiari-like malformation. The dog has idiopathic facial nerve paralysis. The MRI images were normal for this breed, including the lateral ventricle asymmetry. The vasculature was not seen to be abnormal. Computerised Tomography images were also used in this study. The MRI was a 1.5 Tesla Unit; Siemens Magnetom Essenza. The CT scanner used was a dual slice CT scanner by Siemens Somatom Spirit. Further details of each dataset used are given in Table 1. Table 2 lists the software packages (including free alternatives) that were used in the creation of a fully interactive application of canine cranial neurovasculature in this study. Fig 1 demonstrates the workflow used in the creation of this package.

**Table 1:** This table shows the datasets used for analysis of canine vascular neuroanatomy. W: weighted; C: Contrast agent (gadolinium); VIBE: Volume Interpolated Breath-hold Examination; FLAIR: Fluid-attenuated inversion recovery; TE: Echo Time; TR: Repetition Time.

Dataset no.	Type	Sequence	Plane	Slice thickness	Intervals	Region	Number of slices	TE	TR
1	MRI	T2 W	Sagittal	3.50mm	4.2mm	Head	15	97	3590
2	MRI	T2W	Dorsal	3.50mm	4.2mm	Head	18	92	3610
3	MRI	T2W	Transverse	4.00mm	4.400mm	Head	20	85	3810
4	MRI	T1	Transverse	4.00mm	4.400mm	Head	20	13	481
5	MRI	FLAIR	Transverse	4.00mm	4.400mm	Head	20	118	5900
6	MRI	T2*	Transverse	4.00mm	4.40mm	Head	20	12	477
7	MRI	T1Vibe	Transverse	1.00mm	0mm	Head	72	2.38	7.13
8	MRI	T2W	Sagittal	2.50mm	2.75mm	Neck	13	102	3000
9	MRI	T2	Transverse	3.00mm	3.30mm	Neck	18	110	4820
10	MRI	T1+C	Transverse	4.00mm	3.00mm	Head	20	13	481
11	MRI	T1vibe +C	Transverse	1.00mm	0mm	Head	72	2.38	7.13
12	CT		Transverse	1.00mm	1.00mm	Head/Neck	75		

**Table 2:** This table highlights the programmes used for the creation of the digital anatomy. It demonstrates the programme used, where it is produced, the reason for its use in the study and the availability of it, as well as free alternatives that can be considered.

Software	Company	Function	Licensing	Free alternatives
Amira 5.6.0 (*www.fei.com*)	FEI Visualisation Sciences Group; MA, USA	Medical Visualization, Image Processing, Geometry extraction from Volumetric Dataset (i.e MRI, CT,PET)	Academic license	3D Slicer (*https://www.slicer.org/*)
Autodesk 3DS Max 2015 (*www.autodesk.com*)	Autodesk; CA, USA	Modeling, Animation and rendering	Academic license	Blender (*https://www.blender.org/*)
Autodesk Mudbox 2015 (*www.autodesk.com*)	Autodesk; CA, USA	Painting and Sculpting	Academic license	Blender (*https://www.blender.org/) & Sculptris (http://pixologic.com/sculptris/*)
Unity Pro 3D 5.2. *https://unity3d.com/*	Unity Technologies; CA, USA		Academic license	Unity Personal Edition

### Segmentation

The first step necessary in the creation of a 3D reconstruction of the vasculature of the canine head and brain required the raw MRI and CT data to undergo processing in Amira 5.6.0, a 3D data visualisation software package. The raw data was of sufficient image quality that no pre-processing steps were required, thus segmentation, a pre-requisite of surface model generation, could be carried out immediately. During the process of segmentation, a labelled material was assigned to each pixel belonging to a particular group, for example, all pixels belonging to an individual vessel.

The MRI dataset chosen for a particular segmentation varied depending on the region. However, the majority of the structures were segmented using MRI dataset 7 and 11. These sequences were ideal for segmentation purposes given the 1mm slice thickness and large number of slices.

A combination of manual and semi-automated segmentation techniques were applied, essentially on a trial and error basis since the variability exhibited between vessels (i.e. size, curvature), meant there was no method that was ideal for all vessels.

### Semi-automatic techniques

Semi-automatic segmentation of vessels identified on the MRI dataset, based on the region growing algorithm was performed. Region growing provides the largest connected region of pixels around a selected pixel based on value similarity and spatial proximity. This technique worked well where pixel intensity of the chosen vessel contrasted significantly with the pixel intensity of the surrounding anatomy.

### Manual techniques

Manual segmentation techniques were applied where automatic algorithms failed to adequately represent the structure and course of particular vessels. On a slice-by-slice basis, pixels were assigned manually to a corresponding vessel label. This method required altering the MRI dataset contrast to help the visual identification of structures. In parallel, references such as MRI atlases and textbooks were consulted to confirm the correct labelling of vessels [24,25]. The segmentation technique used for each vessel can be seen be seen in Table 3.

**Table 3:** Name of vessel and nature of segmentation applied.

Structure	Type of Segmentation
Internal ophthalmic artery	Manual
Internal ethmoidal artery	Manual
Rostral cerebral artery	Manual
Middle cerebral artery	Manual
Caudal cerebral artery	Manual
Caudal communicating	Manual
Rostral cerebellar artery	Manual
Caudal cerebellar artery	Manual
Basilar artery	Semi-automatic
Internal carotid artery	Manual/Semi-automatic
Dorsal sagittal sinus	Semi-automatic
Straight sinus	Semi-automatic
Transverse sinus	Semi-automatic
Cavernous sinus	Semi-automatic
Ventral petrosal sinus	Semi-automatic
Dorsal petrosal sinus	Manual
Sigmoid sinus	Manual
Temporal sinus	Semi-automatic
Skull	Semi—automatic

The majority of vessel segmentations were carried out using dataset 7 and dataset 11. The vessels could clearly be identified in the transverse slices appearing as white circular regions, with the exception of those that exhibited abrupt changes in direction. In cases such as these, the regions of white representing the vessels would appear more linear than circular. Segmentation of the brain was also performed using dataset 11. It was clearly distinguishable from the surrounding anatomy by the black outline of the skull. Dataset 12 was used to segment the skull and vertebrae given CT data is the preferred method for visualising bone.

### Surface generation

**Fig 2:**
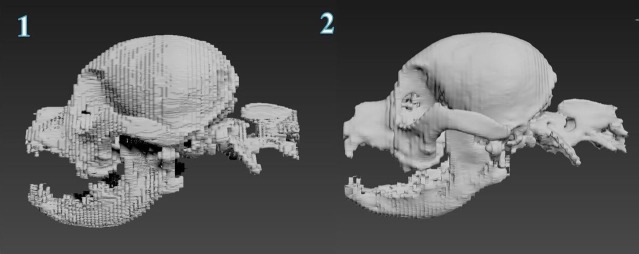
This demonstrates the effects of smoothing in Amira. Image 1 shows the surface generated with no smoothing, and image 2 shows the surface generated with unconstrained smoothing.

On completing the segmentation of the structures, a polygonal surface model was generated based on the results. It was achieved using marching cube, a robust and fast surface reconstruction algorithm, ensuring that no holes appeared on the resulting geometry. In complement, unconstrained smoothing was applied to the entire generated surface. On the one hand, this resulted in enhancement of the final rendering of large structures such as the skull (Fig 2). On the contrary, geometries representing structures with small diameter such as arteries and blood vessels were not smoothed, as unconstrained or constrained smoothing resulted in a major loss of details (Fig 3.1–3.3). Therefore, it was decided to take the unsmoothed arteries and veins forward for post surface processing on modelling and sculpting platforms which provide high degree of control.

**Fig 3:**
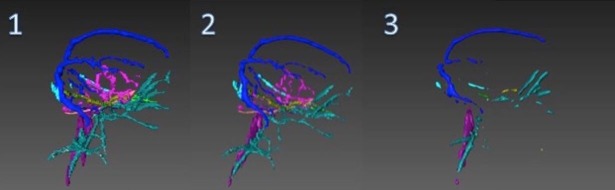
This demonstrates the effects of smoothing in Amira. Image 1 shows the surface generated with no smoothing. Image 2 shows the surface generated with constrained smoothing. Image 3 shows the surface generated with unconstrained smoothing.

### Post processing

The issues resulting from surface generation in Amira 5.6, were addressed during post-processing of the model. First, as the MRI data cropped part of the cerebrum and cerebellum, the rostral aspect of the cerebrum and the caudal aspect of the cerebellum could not be created as surfaces from the 3D surface generation. This meant an approximation had to be created, based on diagrams and photographs from textbook resources. This was achieved in Mudbox, a digital painting and sculpting software package, using the ‘Bulge’ tool to inflate the surfaces that required modelling. The ‘Flatten’ and ‘Smooth’ process was then used to mould the new surfaces into the required shape. This process was also required for the skull, since similar issues regarding the CT dataset was encountered. Despite the smoothing applied on the geometry resulting from surface reconstruction in AMIRA 5.6, further improvements were necessary in Mudbox to work out the blocky aspect of the geometry (Fig 2).

**Fig 4:**
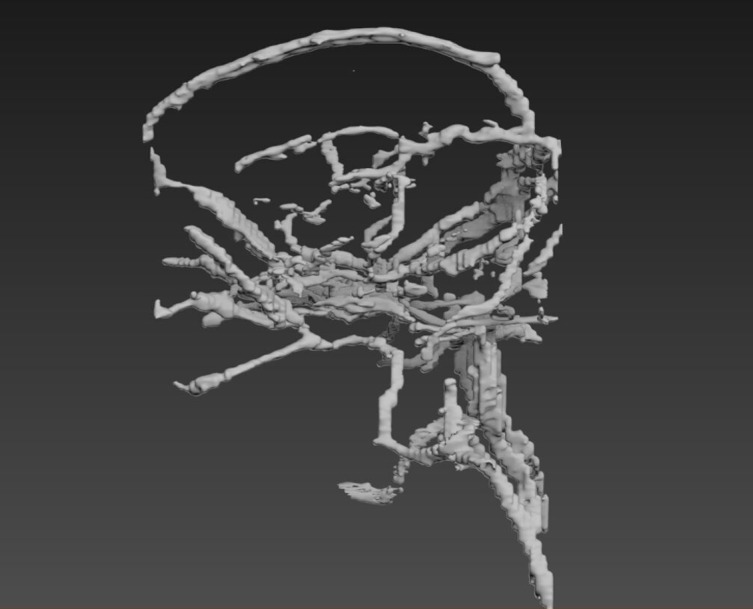
This highlights how the 3D model of the vasculature generated in Amira is “blocky”.

**Fig 5:**
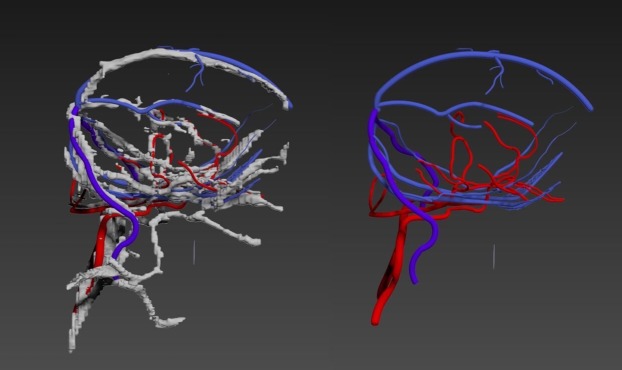
This figure demonstrates the post-processing that was applied in 3DS Max. The image on the left shows the original model generated in Amira being used as a template for the new model composed of cylinders. Image 2 shows the re-modelled appearance of the cerebral vasculature.

Second, as the 3D model of the vasculature generated in AMIRA 5.6 had not been smoothed, the geometry appeared blocky (Fig 4). Moreover, some vessel sections were missing where manual segmentation could not be achieved properly due to lack of resolution of the MRI dataset. To resolve this, the model was imported into 3DS Max to undergo further editing and refinement. Attempts to smooth the existing model using modifiers within 3DS Max proved to be unsuccessful, thus an alternative method had to be investigated. This involved using the existing 3D model as a template to create a new model composed of cylinders. To recreate the natural curvature of blood vessels, a bend modifier was applied to each individual cylinder ensuring the new model was as closely replicated to the original model as possible. The cylinders were then bridged together producing a seamless transition from cylinder to cylinder (Fig 5). Once this process was complete, the new model was saved and exported as an Obj. file, the format required for importing into Unity.

### Interactive application design

An interactive application was created using Unity 3D. This was intended to act as a platform to display the 3D model, supply additional information to the end-user and provide interactive features. In the creation of the interactive application a main menu option was the first to be created. From this, there were two options–a “learn scene” and a “quiz scene”. From the “learn scene”, there were two options–“arterial system” and “venous system”.

#### ‘Learn’ scene

The ‘Learn’ scene was the first scene introduced to the user upon selecting the ‘Learn’ button and this contained the imported 3D reconstruction of the brain and vasculature. Several C# scripts were developed to support meaningful interaction with the model:

1. to allow 360° manipulation around the model using the mouse interface;
2. to return the names of vessels to label geometries on screen upon selection;
3. to highlight vessels on selection in order to make them more evident to the user, and;
4. to display information on selection inside a pop-up panel.

In terms of user interface, a panel displaying the list of arteries and veins/sinuses was created. This panel was designed such that it could be enabled/disabled at the user’s discretion by clicking the title of each list, ‘Arteries’ or ‘Veins’. The vessel names contained within the list were also made interactive. This was achieved using buttons, customised to appear completely transparent except for the text component. Selection of a particular vessel name activates a colour change to white on the corresponding vessel in the model, which could be reset by selecting the vessel name again.

Buttons to alter various aspects of the model were also provided. This functionality was achieved by assigning each button to activate or deactivate specific anatomical parts i.e. skull. The buttons were also designed to appear darker on inactivation, to clearly indicate the button state to the user. In the case of the displaying and hiding the skull, activation of an additional viewpoint positioned further back from the main viewpoint was required. Given the size of the brain in relation to the skull, the position of the main viewpoint was not suitable to clearly show both the brain and the skull.

To enable movement between scenes a menu panel was created to give the user access to a tutorial based on either the arterial system or venous system of the canine brain, take a multiple choice test or quit the application.

#### Tutorial scenes: ‘Arterial System’ and ‘Venous System’

The tutorial scenes were designed based on the content of the Bachelor of Veterinary Medicine and Surgery (BVMS) anatomy course at the University of Glasgow’s, School of Veterinary Medicine. Using the intended learning outcomes of the lectures on the anatomy of the canine brain vasculature, only relevant information was included. Both tutorials follow a similar format, taking place across a number of scenes. The user is presented with information displayed in a panel set to the left hand side of the screen, as well as a modified version of the 3D model that is colour co-ordinated with the specific anatomy described in the accompanying text explanation. The user was allowed to manipulate the point of view around the model as explained above. Buttons were also included to allow the user to move freely between scenes.

#### Quiz scene

Within the ‘Quiz’ scene the user is able to test their knowledge by undertaking a short multiple-choice test. A database of questions with 4 possible answers was built into an array of string. A C# script allowed selecting a set of 10 of those questions and order them randomly. Questions were displayed one at a time in a text panel with each possible answer underneath alongside a selectable toggle button. Progression to the next question was reliant upon the user selecting the correct answer. Instant feedback is provided in the form of text, ‘Correct’ or ‘Incorrect’. At the end of the test, a button enabling the user to return to the ‘Learn’ scene becomes visible. Once the scenes had been created, the application was built for a Windows platform, to be displayed at a resolution of 1024 x 768.

## Results

### 3D model development outcomes

**Fig 6:**
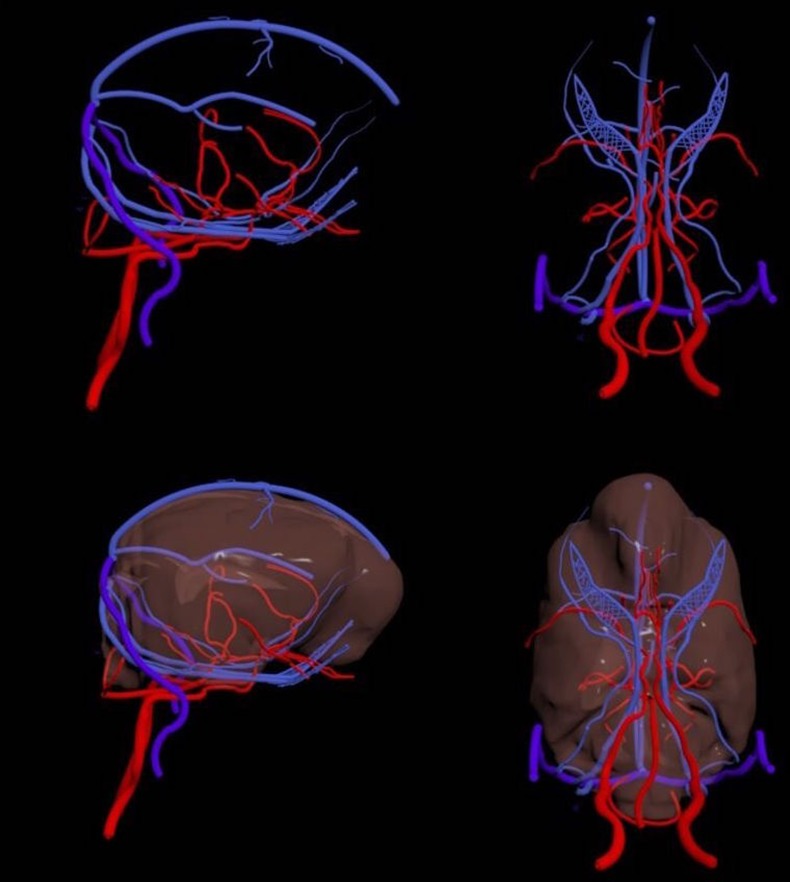
This demonstrates the final development outcome of 3D model reconstruction of the vasculature and brain. The arterial system is shown in red and the venous/sinus system in blue.

Image segmentation, surface generation and post-processing of the model resulted in the development of a 3D reconstruction of the canine skull, brain and associated vasculature. The anatomical structures displayed in the model are highlighted in Table 4 and the vasculature can be seen in Fig 6.

**Table 4:** Structures segmented fro MRI and CT datasets.

**Arteries**	Internal Ethmoidal Arteries
	Internal Ophthalmic Arteries
	Rostral Cerebral Arteries
	Middle Cerebral Arteries
	Caudal Cerebral Arteries
	Caudal Communicating Arteries
	Rostral Cerebellar Arteries
	Caudal Cerebellar Arteries
	Basilar Artery
	Internal Carotid Arteries

**Veins/Sinuses**	Dorsal Sagittal Sinus
	Straight Sinus
	Transverse Sinus
	Cavernous Sinus
	Ventral Petrosal Sinuses
	Dorsal Petrosal Sinuses
	Sigmoid Sinuses
	Temporal Sinuses
	Maxillary Veins
	Internal Cerebral Vein
	Dorsal Cerebral Vein
	Ophthalmic Plexus
	Great Cerebral Vein

**Bone**	Skull

### Interactive application development outcomes

An interactive application was designed using Unity 3D and was made of four components: an interactive model; separate tutorials for the arterial and venous system of the canine brain; and a multiple-choice quiz.

#### ‘Learn scene’

The interactive model was housed within the ‘Learn Scene’. Its features included 360° rotation, vessels highlighted and labelled on selection along with the appearance of an information panel, tabulated and displaying specific vessel information. This included where the vessel originates from and the region it supplies, or in the case of the venous system, the area drained and where it terminates.

**Fig 7:**
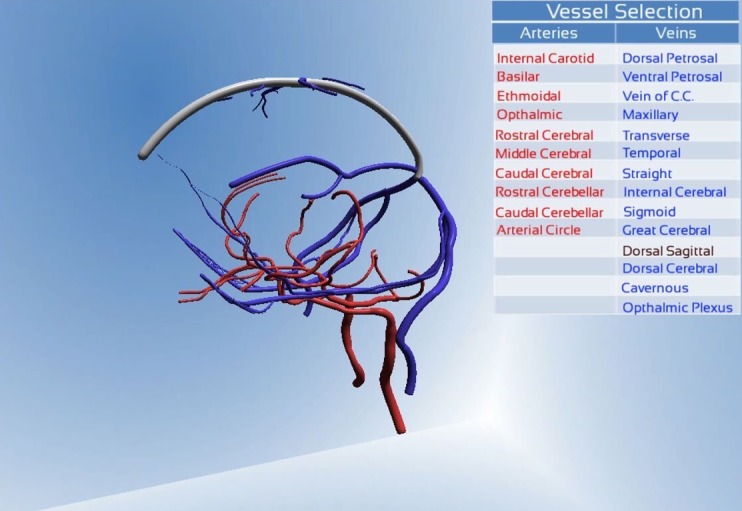
This demonstrates the interactive list feature. The dorsal sagittal sinus was selected in this list, triggering a colour change of the vessel in the model.

A further interactive feature present within the ‘Learn Scene’ was the list of vessel names (Fig 7). Selecting a vessel name activates a colour change on the corresponding model vessel allowing the user to identify specific vessels. This feature was incorporated to provide users with an alternative method of identifying specific vessels, useful where the user requires information but has no prior knowledge of its location on the model.

**Fig 8:**
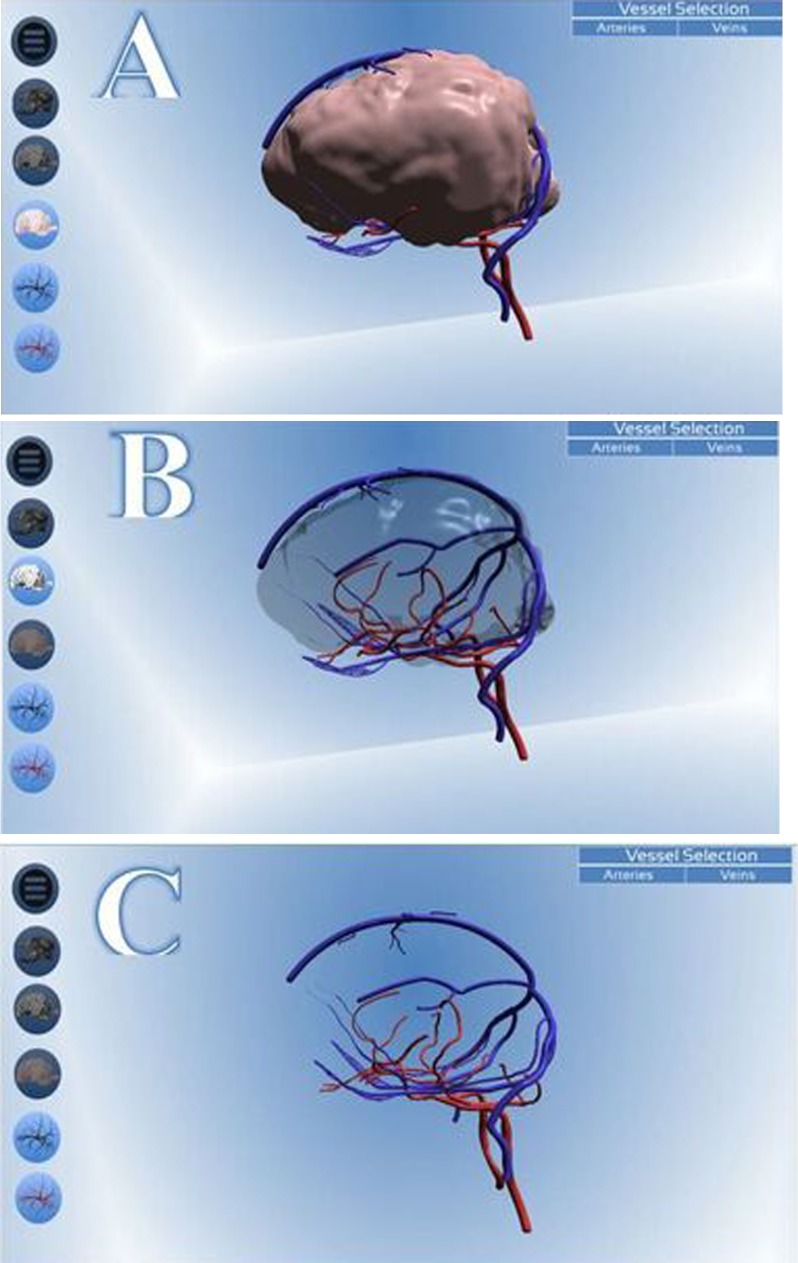
Screenshots demonstrating the options available for displaying the brain. Image A shows the brain being displayed in ‘Opaque’ mode, image B shows the brain displayed in ‘Transparent’ mode and image C shows the model with the brain deactivated completely. The icons corresponding to each function are highlighted. The remaining icons appear darker to indicate they are inactive.

A selection of icons was included allowing different aspects of the anatomy to be displayed or hidden e.g. menu panel, enabling/disabling of the skull, enabling/disabling of the opaque or transparent brain, enabling/disabling of the arterial or venous systems. Three options are available in terms of displaying the brain: 1) an opaque mode to clearly visualise the relationship of the vasculature with the surface of the brain (Fig 8A); 2) a transparent mode to allow the vessels within the brain to be viewed with only its outline visible (Fig 8B); and 3) an option to completely disable this component (Fig 8C). Options are also provided to display the canine skull, only the arterial system or only the venous system.

#### Tutorials: ‘Arterial System’ and ‘Venous System’

From the ‘Learn’ scene, the user had access to both tutorials via the menu icon, the content of which was based on the intended learning outcomes of the BVMS course at the University of Glasgow’s School of Veterinary Medicine.

The ‘Arterial System’ tutorial provides information about the main arteries supplying the brain, the importance of the arterial circle and potential sources of arterial blood to the brain, as well as the function of rete mirabile. In the ‘Venous System’ tutorial the cranial system of venous sinuses is outlined, dealing with the dorsal, ventral and connecting systems separately. Where appropriate, an adapted version of the 3D model is provided with the vessels specifically mentioned in the text colour co-ordinated with the model. 360° rotation is still enabled, however all other interactive features available on the ‘Learn’ scene are not.

#### ‘Quiz’

The final component of the interactive application was the inclusion of a multiple-choice test based on the content of the tutorials. This consisted of 10 questions with 4 possible answers drawn at random from a database of 18. This feature offers the user instant feedback on their understanding of the topic since it demands the correct answer is selected before moving to the subsequent question.

## Discussion

In our study, we have clearly presented a well-defined workflow methodology which has been shown to be successful in creating a fully interactive educational and training resource of canine cerebral neurovasculature. Through the use of commonly available software programmes, the unique workflow presented here, of combining 3D rendering, segmentation and game engine animations has resulted in a realistic anatomical model of the arterial and venous blood supply of the canine brain. This has been achieved through the use of actual MRI and CT data of a Cavalier King Charles Spaniel. Therefore, we have demonstrated, through our workflow, how an innovative and unique training programme can be created to hopefully enhance our undergraduate veterinary students’ understanding of a complex field of anatomy, through a simple and engaging format.

Veterinary anatomy represents a major component of the BVMS course. It is the foundation on which clinical practice is built, providing students with an understanding of animal structure that will support examination of patients, the formation of diagnosis and communication of such findings to owners and other professionals [28,29]. Over the past 20 years, anatomical education has evolved. The value of a multi-modal approach has become widely accepted and teaching no longer relies solely upon the lectures and dissection classes traditionally prescribed [30]. In modern teaching, a typical veterinary anatomy course will incorporate models, medical imaging and computer-assisted learning, among other resources facilitated by new media, to overcome issues that have been associated with the traditional veterinary curriculum [31].

The anatomy of the vascular network supplying the head and brain has been identified as a specific aspect of neuroanatomy that is often under-represented however, yet its role in veterinary medicine is critical [32]. Although true of any region of anatomy, insufficient knowledge inevitably translates into diagnostic errors, subsequently affecting judgement in therapeutic decisions [33]. Furthermore, a sound working knowledge of vascular anatomy carries even more significance in the surgical field where awareness of arterial and venous structures is paramount during neurosurgical interventions to avoid unnecessary and potentially life threatening bleeding [34].

The current methods used to communicate and present the complex arrangement of vasculature often falls short in undergraduate neuroanatomy training [34]. 2D representations of 3D objects unavoidably lead to the loss of certain spatial information, whilst cadaveric dissection/prosection can be hindered by the presence of other structures. Corrosion casts have also traditionally offered great insight into the complicated spatial arrangements of vessels, with current methodologies yielding exceptionally high levels of detail, however with the dissolution of the soft tissue, difficulties can arise relating the cast to its surrounding structures [35].

Given these inadequacies, vascular anatomy in particular can be a challenging topic to grasp using the traditional teaching resources. When combined with the visualisation issues so often expressed by students, it is apparent that this is an aspect of the veterinary anatomy course that could greatly benefit from the use of modern alternatives.

In the early 1990s, the first steps were taken towards the adoption of computer aided learning into veterinary education. An early example of this work was carried out at the Department of Clinical Veterinary Medicine at the University of Cambridge [36]. This initiative was spurred by the advances in computer technology of the time such as the ability to display still images of photographic quality, display video and play high quality sound. As part of this project, a tutorial, question and answer program, digital lecture and a case simulation were included, features typical of first generation computer aided learning programs in veterinary medicine. Notably, this work did not make use of 3D modelling, perhaps due to the apparent lack of whole animal 3D reconstructions.

One of the earliest attempts at digitising 3D animal anatomy was described in the ‘Whole Frog Project’, the goal of which was to show the feasibility of sophisticated image based applications in high school level biology. Based on MRI data and photography of cryosections, image segmentation was carried out to produce a 3D model of a frog that was accessible via an online ‘virtual dissection kit’ [37].

In terms of resources developed for university level education, anatomic reconstructions of the rat brain and equine metacarpophangeal joint were also developed in the 1990s [17,18]. Based on the same protocols as the ‘Whole Frog Project’, 3D models of the respective species anatomy were created, with the intention of future integration within educational platforms.

The ‘Visible Animal Project’, represented the first whole animal 3D model developed specifically for veterinary education, nearly a decade after the introduction of computer aided learning. Again, processing of CT and MRI and cryosection data was used to yield a high-resolution 3D database of the dog, in particular the musculoskeletal system of the head, neck and trunk, major vessels and organs [19]. While this model gave an accurate and realistic overview of these areas, it lacked detail of the cranial anatomy of the dog, which has been identified as an important yet notoriously difficult region for students to understand.

This particular issue was recognised by Linton et al. [38], thus ‘Virtual Canine Anatomy: The Head’ was developed and incorporated into a first year veterinary dissection laboratory at Colorado State University. This program was created using an alternative method to those previously discussed, instead using specimen photographs and QuickTime Virtual Reality (QTVR). A series of photographs were displayed showing the progressive stages of a dissection that allow the students to click on anatomical structures to display the associated description. In the strictest sense, this program does not utilise a 3D model, instead alternate 2D views of the structure are available that can be rotated to give the illusion of a 3D object, using QTVR. Nevertheless, the value of such a resource is undeniable, and evaluation of the program revealed its use increased student confidence and efficiency in actual dissection laboratories. A limitation that could be associated with this particular program however stems back to the original issues surrounding cadaveric dissection, in that visualising spatial arrangements of structures such as vasculature can be difficult in a complex anatomical scene.

While computer aided learning applications in veterinary medicine have certainly come a long way since those introduced in the early 90s, there is still no interactive, anatomically accurate 3D model of the full dog, to our knowledge. Indeed, there is not a clear interactive 3D toolset enabling the spatial arrangement of vasculature of the brain to be fully understood.

As such, this fully interactive 3D anatomical training package we have created here in our workflow methodology should help resolve many of these issues. However, it certainly would have to be tested formally on the end user–undergraduate veterinary students.

### Limitations

Against the success of the development of the workflow methodology presented here, there do still exist some limitations. Some elements of this process resulted in manual segmentation being adopted. As such, this is a very time consuming process which can result in a disproportionate amount of time being taken on some areas of creation of the package. Although some structures could be fully automatically segmented, being able to do so across all datasets would help to alleviate this problem. That will depend on the advancement of the software packages and their ability to process datasets from complex algorithms.

Moreover, neither automatic nor manual segmentations guaranty an errorless reconstruction of the anatomy as both processes usually strongly rely on the quality of the images that composed the dataset. For instance, poor Signal to Noise Ratio (SNR) from images created from MRI may eventually hamper the accurate reconstruction of anatomical structures.

In addition to the segmentation as a challenging process, the MRI dataset provided failed to image the entire brain. Rather than discarding this dataset, we optimised the 3D modelling process to construct an approximation of those missing regions. This could have impacted on the anatomical accuracy of the canine brain, but as the primary aim was to provide a representation of the vasculature, we feel that this was minimal on impacting the digital reconstructions thus created.

### Future work

Given the intended purpose of this interactive application, as a supplementary resource for veterinary anatomy, validation of this model through user testing would undoubtedly be the next logical step. Furthermore, an investigation focussing on the impact of this application on students with low spatial ability would be of particular value in substantiating its place in the veterinary curriculum.

The methodology utilised in this project can however be considered successful. While several steps required refinement, for example the lengthy segmentation process, there is no reason why this process could not be used to create similar works for different species. The cerebral vasculature and blood supply to the brain is a highly variable region of anatomy amongst different species, especially regarding the internal carotid artery [35]. An opportunity to explore and compare such variation in vascular networks would be undoubtedly be useful in veterinary education.

Further enhancement of the existing model by including additional anatomical detail could also be a future line of enquiry. In particular the 3D model developed in this study could benefit from the addition of a detailed segmentation of the brain. This would allow blood supply from a particular vessel to be delineated more precisely on the visualisation for full distribution to a micro-vascular level.

## Conclusion

The aim of this research was to clearly demonstrate how widely available software programmes could be used in the creation of a fully interactive 3D package to enhance understanding of the neurovasculature of canine cerebral anatomy. We have therefore shown that it is possible to create an interactive educational and training package for this purpose. Certainly, further validation and engagement with the end user (students), as well as additional veterinary clinicians would be needed to fully assess the impact of its educational application. However, through this process, we have now created a path for future veterinary educators and students to become involved in creating tailor made packages for their students and professionals alike. Indeed, this is not limited to veterinary practitioners and their students. This type of workflow methodology can be used across the medical, scientific and allied health professionals fields in construction of educational and training materials based on initial CT and/or MRI data. It also acts as a platform to use other types of imaging modalities and clearly establishes a defined workflow methodology which can be used in creation of unique and engaging ways to engage the learner and the teacher alike.
